# Systematic review of effect of community-level interventions to reduce maternal mortality

**DOI:** 10.1186/1471-2393-9-2

**Published:** 2009-01-20

**Authors:** Elaine Kidney, Heather R Winter, Khalid S Khan, A Metin Gülmezoglu, Catherine A Meads, Jonathan J Deeks, Christine MacArthur

**Affiliations:** 1 Dept of Public Health and Epidemiology, University of Birmingham, Edgbaston, Birmingham, B15 2TT, UK; 2 Academic Unit, Birmingham Women's Hospital, University of Birmingham, Birmingham, UK; 3 UNDP/UNFPA/WHO/World Bank Special Programme of Research, Development and Research Training in Human Reproduction, WHO, Geneva, Switzerland

## Abstract

**Background:**

The objective was to provide a systematic review of the effectiveness of community-level interventions to reduce maternal mortality.

**Methods:**

We searched published papers using Medline, Embase, Cochrane library, CINAHL, BNI, CAB ABSTRACTS, IBSS, Web of Science, LILACS and African Index Medicus from inception or at least 1982 to June 2006; searched unpublished works using National Research Register website, metaRegister and the WHO International Trial Registry portal. We hand searched major references.

Selection criteria were maternity or childbearing age women, comparative study designs with concurrent controls, community-level interventions and maternal death as an outcome. We carried out study selection, data abstraction and quality assessment independently in duplicate.

**Results:**

We found five cluster randomised controlled trials (RCT) and eight cohort studies of community-level interventions. We summarised results as odds ratios (OR) and confidence intervals (CI), combined using the Peto method for meta-analysis. Two high quality cluster RCTs, aimed at improving perinatal care practices, showed a reduction in maternal mortality reaching statistical significance (OR 0.62, 95% CI 0.39 to 0.98). Three equivalence RCTs of minimal goal-oriented versus usual antenatal care showed no difference in maternal mortality (1.09, 95% CI 0.53 to 2.25). The cohort studies were of low quality and did not contribute further evidence.

**Conclusion:**

Community-level interventions of improved perinatal care practices can bring about a reduction in maternal mortality. This challenges the view that investment in such interventions is not worthwhile. Programmes to improve maternal mortality should be evaluated using randomised controlled techniques to generate further evidence.

## Background

There are 529,000 maternal deaths each year, 99.6% of these in developing countries [1] where health care provision is rudimentary. The lifetime risk of maternal death was estimated in 2000 as 1 woman in 16 in sub-Saharan Africa and 1 in 46 in south-central Asia, compared to 1 in 2800 in developed regions [1]. Most maternal deaths are preventable. Despite numerous initiatives by governments, WHO, UNICEF, UNFPA, World Bank and others, and a Millennium Development Goal (MDG 5) to reduce maternal mortality by three-quarters by 2015, there has been little improvement [2]. In some areas mortality has worsened [3]. Whether intervention programmes have been simple or complex, few have robustly assessed their effect on maternal deaths. Indeed, some authors have advocated that due to the large population sizes needed and the difficulty in obtaining reliable data, maternal mortality is an unsuitable indicator for assessing safe motherhood initiatives [4].

Meeting MDG 5 is likely to need a combination of strategies, including those at community-level, utilizing best evidence on antenatal, intrapartum and postpartum care. Complete access to facility based obstetric care may be best practice [5] but is likely to be many years away and even then will not be acceptable or accessible to all [6,7]. It is plausible that community-level interventions could improve outcomes by increasing the standard of out-of-facility care through training or education, or by improving care-seeking behaviour and access to facility level care. However, opinions diverge as to whether investment in community-level interventions is worthwhile [5,8]. We undertook a systematic review to assess the effectiveness of community-level interventions to reduce maternal mortality.

## Methods

### Sources

We searched the following electronic databases: MEDLINE, EMBASE, CINAHL, BNI, CAB ABSTRACTS, IBSS, Cochrane (Central, DARE and NHS EED and Systematic Reviews), Web of Science (SCI-expanded and SSCI), LILACS and African Index Medicus from database inception or at least from 1982 to June 2006 for terms including maternal mortalit*, communi* intervention*, participat*intervention*, midwi* and birth* attend* (see Additional file 1), and used Reference Manager 11 software  to keep track of citations identified. We followed up references in published papers, contacted leading authors, and hand searched major relevant journals up to July 2007. We searched for unpublished work using the National Research Register website , 2006 issue 2, searched 30 June 2006), metaRegister , searched 30 June 2006) and the WHO International Trial Registry portal , searched 24 May 2007). No language restrictions were applied.

### Study selection

We developed a protocol using recommended methods for collating data from randomised controlled trials and cohort studies with concurrent controls following QUOROM and MOOSE checklists [9-12]. Selection criteria were general maternity populations or women of childbearing age (15 to 49 years) taking part in a community-level intervention. We defined a "community-level" intervention as one which is either accessed locally at the woman's home, village, school or local clinic, or delivered by any person within the community, including health personnel or lay individual. Studies conducted in primary care settings designed to provide normal pregnancy and childbirth services and refer women with complications to higher levels of care were eligible for inclusion. All eligible studies had to report on maternal deaths and have controls of concurrent comparable populations experiencing either "usual care", including hospital based care, or other community interventions. We used the ICD10 definition of maternal mortality (death of a woman while pregnant or within 42 days of termination of pregnancy, irrespective of the duration and site of the pregnancy, from any cause related to or aggravated by the pregnancy or its management but not from accidental or incidental causes). We excluded studies of pharmacological and nutritional interventions since these have already been the subject of other systematic reviews. We also excluded studies on non-general maternity or childbearing age populations, such as women with a particular disease. No language restrictions were applied.

Two authors (EK and CMacA) independently assessed titles and abstracts of all potential references for eligibility and retrieval of full papers. In cases of uncertainty the original papers or raw data were obtained or authors contacted to reach a decision about eligibility after consideration of the published information. All retrieved papers were assessed in duplicate (by EK and HW), using checklists of inclusion and exclusion criteria.

Two authors (EK and either CM or KSK) independently quality assessed studies and extracted data using a piloted data extraction form. We defined study quality as the extent to which design, methods, execution and analysis minimised bias in assessment of effectiveness, focusing on internal validity [9]. We classified studies as high, medium, low (or unclear) quality with respect to selection, performance, measurement and attrition biases as shown in Table 1. Individual studies were described by study type, intervention, location and date, numbers taking part, population denominator (eg pregnancy or live birth) and study quality (Table 2). Any discrepancies were resolved by discussion with additional authors (CMacA and HW).

**Table 1:** Quality assessment criteria

	**High quality**	**Medium quality**	**Low quality**
**1. Selection bias:**	Studies with randomisation, allocation concealment, similarity of groups at baseline	RCTs with some deficiencies in randomisation e.g. lack of allocation concealment, or non-randomised studies with either similarities at baseline or use of statistical methods to adjust for any baseline differences	Non randomised, with obvious differences at baseline, and without analytical adjustment for these differences.

2. Performance bias:*	Differed only in intervention, which was adhered to without contamination, groups were similar for co-interventions or statistical adjustment was made for any differences	Confounding was possible but some adjustment was made in the analysis	Intervention was not easily ascertained or groups were treated unequally other than for intervention or there was non-adherence, contamination or dissimilarities in groups and no adjustments made.

**3. Measurement bias:**	Outcome measured equally in both groups, with adequate length of follow-up (i.e. at least 6 weeks postpartum), direct verification of outcome, with data to allow calculation of precision estimates.	Inadequate length of follow up or length not given	Inadequate reporting or verification of maternal mortality or differences in measurement in both groups

**4. Attrition bias:**	No systematic differences in withdrawals between groups and with appropriate imputation for missing values		Incomplete follow-up data, not intention-to-treat analysis or lacking reporting on attrition

*Blinding was not a quality assessment as blinding of participants or caregivers to intervention types was not possible

We extracted data to generate maternal mortality rates for the intervention and comparator arms of the included studies. For cluster randomised trials we computed the design effect from data presented in the study reports (intra-class correlation coefficients, cluster adjusted estimates and confidence intervals) using STATA 9.2  and adapted sample sizes and numbers of events to make appropriate allowance for clustering [13]. Where such data could not be found, we computed a design effect using the mean intra-class correlation coefficient from the trials in which they were computable. We summarised results of individual studies as odds ratios (OR) and confidence intervals adjusted for clustering, which we pooled using the Peto method validated for meta-analysis of rare events [14]. We considered results of randomised trials and cohort studies separately. Meta-analysis were only considered for studies without a high risk of bias, which were then stratified according to the purpose of the intervention.

## Results

**Figure 1:**
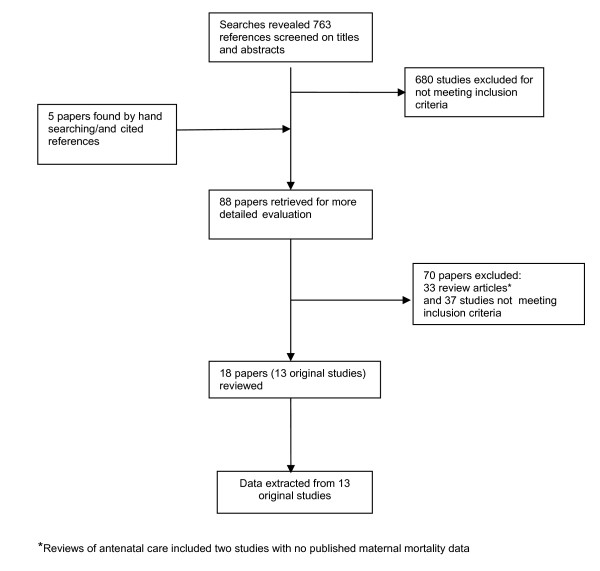
**Study selection process**.

We identified 768 relevant references, of which 13 studies met our inclusion criteria (Figure 1). The quality of the studies was extremely variable (Table 2). Eleven of the studies involved 118,467 maternities (pregnant women, pregnancies, deliveries or live births). Two studies had no data on participant numbers. We found five cluster randomised controlled trials, of which the trials by Jokhio [15] and Munjanja [16] had large numbers of participants but few clusters. Of the eight non-randomised studies, only one scored "high" and two "medium " in any of the four quality assessments. The rest scored either "low" or unclear"

**Table 2:** Characteristics and maternal mortality outcomes of included studies

**Study**	**Design**	**Intervention**	**n**	**Quality assessment**	**MMR Intervention**	**MMR Control**	**OR (95%CI)**
Manandhar 2004[17] rural Nepal	Cluster RCT 24 clusters	Facilitator-led women's groups to improve perinatal care practices plus health-service strengthening vs. usual care plus health-service strengthening	28,931 women of childbearing age 6,714 pregnancies 6,165 live births	1:medium; 2: low; 3: medium ; 4: high	69	341	0.28 (0.09 to 0.82)

Jokhio 2005[15] rural Pakistan	Cluster RCT 7 clusters	TBA training and health service integration, issue of sterile delivery kits vs. usual care	19,557 pregnant women 19,524 deliveries	1:high; 2:high; 3 high; 4: high	268	360	0.74 (0.45 to 1.23)

Munjanja 1996[16] Harare, Zimbabwe	Cluster RCT 7 clusters	Intervention: fewer, but goal-oriented antenatal visits vs. standard "westernised" antenatal care	15,994 low risk pregnancies 15,532 deliveries	1:medium; 2: medium; 3:medium; 4: low	64	82	0.78 (0.23 to 2.61)

Villar 2001 [20] Argentina, Cuba, Saudi Arabia, Thailand	Cluster RCT 53 clusters	Intervention: fewer, but goal-oriented antenatal visits vs. standard "westernised" antenatal care	24,526 low risk pregnant women 22,793 single births	1:high; 2:high 3: medium 4:high	60	54	1.11 (0.37 to 3.29)

Majoko 2007[19] rural Zimbabwe	Cluster RCT 23 clusters	Intervention: fewer, but goal-oriented antenatal visits vs. standard "westernised" antenatal care	13179 pregnant women	1:medium; 2:high; 3: low; 4: high	60	31	1.90 (0.38 to 9.43)

Ackermann-Liebrich 1996[28] Switzerland	Prospective cohort study with nested matched pairs	Women opting for home vs. hospital birth in "westernised" setting	874 pregnant women 857 deliveries	1:low; 2: low; 3 :not applicable; 4:low	0	0	

de Bernis 2000, Dumont, 2002[29,30] Senegal	Prospective survey of two cohorts	Women in Kaolack delivered mainly by TBAs in district birth centres vs. women in St Louis delivered mainly by midwives in hospital	3,777 pregnant women 3,689 deliveries 3,476 live births	1:low; 2:medium; 3:high; 4:unclear	874	151	5·84 (1·66 to 20·53)

Greenwood 1990[31] rural Gambia	Prospective cohort	TBA training, village health worker support and obstetric pack vs. no additional care	1,963 pregnancies	1:low; 2:medium; 3:medium; 4:unclear	1051	963	1·09 (0·43 to 2·75)

Fauveau 1991; Maine 1996[23,32] rural, Bangladesh (Matlab)	Prospective cohort 1987–1989	Midwives working with community health workers and TBAs to attend home births, manage obstetric complications and accompany referral cases to project clinic vs. routine care (not described) plus access to project clinic	9,630 live births	1:low; 2:low; 3:unclear; 4:unclear	136	388	0·35 (0·13 to 0·93)

Ronsmans 1997[24] rural Bangladesh (Matlab)	Prospective cohort 1990–1993	Access to above Matlab Intervention vs. "routine care"	24,059 live births	1:low; 2:low; 3:unclear; 4:unclear	239	289	0·83 (0·5 to 1·36)

Foord 1995; Fox-Rushby & Foord 1995, 1996. [33-35] The Gambia	Prospective cohort	Early identification of pregnant women by trained TBAs, mobile antenatal unit to treat anaemia and infections; referral/transfer for obstetric emergency treatment; low-cost insurance scheme to pay for treatment vs. care by TBAs with minimal tertiary facilities	1,059 women delivering	1:low; 2:low; 3:low; 4:unclear	126	693	0·43 (0·02 to 1·55)

Xu 1995[36] China	Prospective cohort	Reorganisation of maternity care to include better clinical governance, education and training of staff, and some community education	unknown	1:low; 2:unclear; 3:low; 4:unclear	37	93	0·39

Zhang 2004[37] China	Cohort: Complex stratification of "randomly selected" project and matched non-project areas	Maternal and child health providers at grass roots level given two weeks theory training; some also given one month clinical skills training	unknown	1:low; 2:unclear; 3:low; 4:unclear	53	52	1·06

Quality assessment codes: 1 = selection bias; 2 = performance bias; 3 = measurement bias; 4 = attrition bias

TBA: Traditional birth attendant

MMR: maternal mortality ratio (deaths/100,000 live births)

None of the trials was powered to assess maternal mortality, although one trial did show a significant reduction based on small numbers of deaths [15]. Of the randomised controlled trials, two evaluated interventions to improve perinatal care practices [15,17]. Both aimed to educate lay birth attendants in awareness of basic concepts of maternal and neonatal care, reduce unsafe delivery practices, and increase referral where there may be problems [15,17,18]. Both provided adequate information to estimate the impact of the cluster design on maternal mortality outcomes. No adjustment for the cluster design was required for maternal mortality, as the intra-class correlation coefficient (ICC) was estimated to be zero. Three trials with equivalence hypotheses assessed reduced frequency but goal-oriented antenatal care models [16,19,20]. None of these trials adjusted for cluster effect for maternal mortality (for the meta-analysis we presumed that they also would have an ICC of zero, and no adjustment for clustering was made).

The outcomes of all included studies are shown in Table 2. The non-randomised studies were of insufficient quality to draw conclusions about their effectiveness.

**Figure 2:**
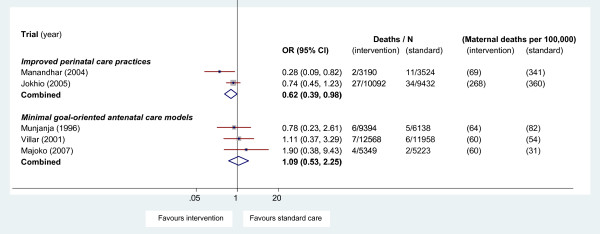
**Effect of improved perinatal care and minimal goal-oriented antenatal care models on maternal mortality**.

For the studies with low risk of bias, separate meta-analyses were undertaken for the two types of intervention (Figure 2). Interventions to improve perinatal care practices showed a statistically significant difference in maternal mortality favouring treatment (OR = 0.62; 95% CI 0.39 to 0.98, p = 0.042). The meta-analysis to examine whether minimal targeted antenatal care was non-inferior to standard care showed no difference in maternal mortality (OR = 1.09; 95% CI 0.53 to 2.25, p = 0.81), although the confidence interval was wide.

## Discussion

This systematic review found a paucity of comparative studies of community-level interventions measuring maternal mortality. Only the RCTs provided reliable evidence. Meta-analysis of the interventions aimed at improving perinatal care practices, already shown to be effective in reducing perinatal or neonatal mortality, showed a reduction in maternal mortality. This constitutes evidence that is too strong for the effect of community-level perinatal care practices on reducing maternal mortality to be dismissed.

From the outset, we employed a broad definition of "community-level" to include interventions which were accessed or delivered locally in the community. This meant that three studies of antenatal care practices met our inclusion criteria, as they were conducted in local clinics. These trials of minimal but goal-oriented antenatal care, designed to determine whether this model was as effective as standard, more frequent "westernised" care, showed no difference in maternal death. These results confirm and consolidate the findings of the Cochrane review on patterns of antenatal care for low risk pregnancy [21] and the WHO systematic review of routine antenatal care [22], incorporating a more recent trial by Majoko et al [19]. The implications for antenatal care practice are fully discussed in these reviews and will not be repeated here.

This systematic review employed an exhaustive search strategy, with a wide range of databases and searches, enabling us to compile a comprehensive collection of potentially relevant studies reporting maternal deaths. We had considered that inclusion of non-randomised controlled studies could have provided the review with added breadth. Unfortunately, poor quality, in particular high chance of selection bias resulting from lack of randomisation, renders their results highly inconclusive. One of these studies, in Matlab, Bangladesh, represented a major initiative in the attempt to change maternity services to reduce maternal mortality. Community midwives worked with community health workers and traditional birth attendants to attend home-births, detect and manage obstetric complications and refer to higher level care as required. A decrease in mortality was observed (OR 0.35, 95% CI 0.14 to 0.88) which was taken as sufficient evidence to extend the intervention[23]. However, an adjacent comparison area subsequently experienced a similar decrease in mortality, and the improvement in mortality rates in both areas was later largely ascribed to women's self-referral to accessible hospital emergency obstetric care[24]. More recently, other factors have also been postulated, such as safe abortion and family planning to reduce abortion deaths [25]. After 30 years, the Matlab studies have been unable to determine with certainty the effectiveness of interventions.

A limitation of this review was the paucity of eligible randomised trials and the necessity to undertake two meta-analyses because of the two distinct types of intervention. Meta-analysis is a powerful and valid tool for combining rare outcomes, provided that appropriate methods are used which avoid large sample approximations [14]. There were however, inadequate data to test for heterogeneity, which should be considered a secondary issue when pooling rare outcomes [14]. Visually, heterogeneity for the antenatal care models appeared to be low but it is difficult to assess with so few trials.

Another potential limitation arises from the outcome of interest being extremely rare. This review included only studies which stated whether deaths had occurred. This meant that two small studies which did not report maternal deaths [26,27] (one with a death, one without) found in the reviews on antenatal care patterns were not included in the present review. We cannot exclude the possibility that other studies may have known of, but not reported, maternal deaths.

The interventions were so similar in the three reduced but goal-oriented antenatal care trials that, the decision to pool their data was obvious. Combining results of both trials of improved perinatal health care practices may initially seem counter-intuitive. The intervention in Nepal was focussed on facilitator-led women's groups [17,18] and the intervention in Pakistan on linkage and training of traditional birth attendants [15]. However, we believe a meta-analysis of these trials to be appropriate, since the interventions in both aimed to improve the standard of perinatal care, by increasing hygienic practices and integrating modestly improved primary health care services, as reflected in process outcomes for both trials. Both trials were in resource-poor rural settings.

The evidence from this review, albeit based on only two trials and both in rural Asia, suggests that community-level interventions to improve perinatal care practices can also reduce maternal mortality. Few would contest the importance of continuing to establish maternity services which allow women to deliver in a health facility and have timely access to comprehensive obstetric emergency care. These services must provide appropriate evidence-based care, be adequately resourced and acceptable to women. In many countries with high maternal mortality, the strategy of intra-partum care based in health centres *"is simply not achievable with current resources and infrastructure, and without other evidence-based options, countries could be left without adequate guidance about how to proceed*" [8]. Alongside strengthening facility based care, therefore, there is a need to develop and properly evaluate community-level strategies to improve maternal health outcomes, including mortality, especially in rural areas. Such strategies are inevitably complex and multifaceted. Thus process outcomes that describe how the strategy is implemented (eg distribution and use of clean delivery kits, use of sterile blade to cut the cord, whether birth attendant washed her hands) and the participants' experience (eg referral to higher level care, type and place of delivery) are necessary to understand "how" the intervention works and will improve the generalisability of the results.

The studies in Nepal and Pakistan have clearly shown that it is possible to design, implement and evaluate large scale interventions using randomised controlled designs. These trials have already produced valid policy-relevant evidence to show that neonatal and perinatal mortality can be reduced by community-level interventions to improve perinatal care practices. Such interventions, directed at improving maternity services, are likely to influence both maternal and perinatal outcomes but many studies are primarily generated and evaluated from one perspective only. Debate as to whether perinatal mortality can serve as a proxy for maternal mortality [15] may be less appropriate than directing concerted efforts to reduce and measure both within maternity services programmes. Many countries and agencies do implement large-scale and expensive programmes which could generate effectiveness evidence by incorporating cluster trial techniques, such as rolling out the programme in a randomised manner ('randomised roll-out'). This type of approach will, in the medium to longer term, produce evidence on which to base policies on how to achieve significant reductions in maternal mortality.

## Conclusion

In summary, this review found that only RCTs provided reliable evidence. This evidence suggests that community-level interventions that improve perinatal practices can reduce maternal mortality. This finding challenges the view that investment in community-level interventions is not worthwhile, although further studies in different settings and contexts are needed. Study designs now allow for robust evaluation of large-scale programmes. It is imperative that we use randomised techniques to obtain more conclusive evidence of how to achieve the 5th MDG of reducing maternal mortality.

## Competing interests

The study was funded by the University of Birmingham. HRW was joint author of one of the studies under review. We declare that we have no conflict of interest.

## Authors' contributions

HW, CMacA and KSK proposed the original idea for this paper. All authors contributed to the protocol. EK designed and carried out the searches. EK and CMacA screened initial references for retrieval. KSK, CM and EK designed the data extraction form and extracted the data, and CMacA and HW and EK completed the quality table. JD undertook the statistical analysis. EK, CMacA and HW wrote the paper and KSK, MG and JD contributed to it. All authors approved the writing of the text.

## Pre-publication history

The pre-publication history for this paper can be accessed here:



## Supplementary Material

Additional file 1 **Appendix.** Search strategy used in systematic review of community-level interventions to reduce maternal mortality Click here for file

## References

[B1] AbouZahr C (2004). Maternal mortality in 2000: Estimates developed by WHO, UNICEF and UNFPA.

[B2] Starrs AM (2006). Safe motherhood initiative: 20 years and counting. Lancet.

[B3] WHO (2005). The World Health Report 2005 – make every mother and child count.

[B4] Graham WJ, Filippi VA, Ronsmans C (1996). Demonstrating programme impact on maternal mortality. Health Policy Plan.

[B5] Campbell OMR, Graham WJ, on behalf of The Lancet steering group (2006). Strategies for reducing maternal mortality: getting on with what works. Lancet.

[B6] Barnes-Josiah D, Myntti C, Augustin A (1998). The 'three delays' as a framework for examining maternal mortality in Haiti. Soc Sci Med.

[B7] Thaddeus S, Maine D (1994). Too far to walk: maternal mortality in context. Soc Sci Med.

[B8] Costello A, Azad K, Barnett S (2006). An alternative strategy to reduce maternal mortality. Lancet.

[B9] Khan KS, ter Riet G, Popay J, Nixon J, Kleijnen J, Khan KS, ter Riet G, Glanville J, Sowden AJ, Kleijnen J (2001). Undertaking Systematic Reviews of Research on Effectiveness, CRDs Guidance for those Carrying Out or Commissioning Reviews.

[B10] Downs SH, Black N (1998). The feasibility of creating a checklist for the assessment of the methodological quality both of randomised and non-randomised studies of health care interventions. J Epidemiol Community Health.

[B11] Moher D, Cook D, Eastwood S, Olkin I, Rennie D, Stroup D (1999). Improving the quality of reports of meta-analyses of randomised controlled trials: the QUOROM statement. Quality of Reporting of Meta-analyses. Lancet.

[B12] Stroup D, Berlin J, Morton S, Olkin I, Williamson G, Rennie D (2000). Meta-analysis of Observational Studies in Epidemiology: A Proposal for Reporting. JAMA.

[B13] Deeks J, Higgins J, Altman D, Higgins J, Green S (2006). Analysing and presenting results. Cochrane Handbook for Systematic Reviews of Interventions 426 [updated September 2006] Section 8.

[B14] Bradburn M, Deeks J, Berlin J, Localio A (2007). Much ado about nothing: a comparison of the performance of meta-analytical methods with rare events. Stat Med.

[B15] Jokhio A, Winter H, Cheng K (2005). An Intervention Involving Traditional Birth Attendants and Perinatal and Maternal Mortality in Pakistan. New Engl J Med.

[B16] Munjanja SP, Lindmark G, Nystrom L (1996). Randomised controlled trial of a reduced-visits programme of antenatal care in Harare, Zimbabwe. Lancet.

[B17] Manandhar D, Osrin D, Shrestha B, Mesko N, Morrison J, Tumbahangphe K (2004). Effect of a participatory intervention with women's groups on birth outcomes in Nepal: cluster-randomised controlled trial. Lancet.

[B18] Morrison J, Tamang S, Mesko N, Osrin D, Shrestha B, Manandhar M (2005). Women's health groups to improve perinatal care in rural Nepal. BMC Pregnancy and Childbirth.

[B19] Majoko F, Munjanja SP, Nyström L, Mason E, Lindmark G (2007). Randomised controlled trial of two antenatal care models in rural Zimbabwe. BJOG.

[B20] Villar J, Ba'aqeel H, Piaggio G, Lumbiganon P, Miguel Belizan J, Farnot U (2001). WHO antenatal care randomised trial for the evaluation of a new model of routine antenatal care. Lancet.

[B21] Villar J, Carroli G, Khan-Neelofur D, Piaggio G, Gülmezoglu M (2001). Patterns of routine antenatal care for low-risk pregnancy. The Cochrane Database of Systematic Reviews.

[B22] Carroli G, Villar J, Piaggio G, Khan-Neelofur D, Gulmezoglu M, Mugford M (2001). WHO systematic review of randomised controlled trials of routine antenatal care. Lancet.

[B23] Fauveau V, Stewart K, Khan SA, Chakraborty J (1991). Effect on mortality of community-based maternity-care programme in rural Bangladesh. Lancet.

[B24] Ronsmans C, Vanneste AM, Chakraborty J, van Ginneken J (1997). Decline in maternal mortality in Matlab, Bangladesh: a cautionary tale. Lancet.

[B25] Chowdhury ME, Botlero R, Koblinsky M, Saha SK, Dieltiens G, Ronsmans C (2007). Determinants of reduction in maternal mortality in Matlab, Bangladesh: a 30-year cohort study. Lancet.

[B26] Sikorski J, Wilson J, Clement S, Das A, Smeeton N (1996). A randomised controlled trial comparing two schedules of antenatal visits: the antenatal care project. BMJ.

[B27] McDuffie R, Beck A, Bischoff K, Cross J, Orleans M (1996). Effect of frequency of prenatal care visits on perinatal outcome among low-risk women. A randomized controlled trial. JAMA.

[B28] Ackermann-Liebrich U, Voegeli T, Gunter-Witt K, Kunz I, Zullig M, Schindler C (1996). Home versus hospital deliveries: follow up study of matched pairs for procedures and outcome. BMJ.

[B29] de Bernis L, Dumont A, Bouillin D, Gueye A, Dompnier JP, Bouvier-Colle MH (2000). Maternal morbidity and mortality in two different populations of Senegal: a prospective study (MOMA survey). BJOG.

[B30] Dumont A, de Bernis L, Bouillin D, Gueye A, Dompnier JP, Bouvier-Colle MH (2002). Maternal morbidity and qualification of health-care workers: comparison between two different populations in Senegal. J Gynecol Obstet Biol Reprod (Paris).

[B31] Greenwood AM, Bradley AK, Byass P, Greenwood BM, Snow RW, Bennett S (1990). Evaluation of a primary health care programme in The Gambia. I. The impact of trained traditional birth attendants on the outcome of pregnancy. J Trop Med Hyg.

[B32] Maine D, Akalin MZ, Chakraborty J, De FA, Strong M (1996). Why did maternal mortality decline in Matlab?. Stud Fam Plann.

[B33] Foord F (1995). Gambia: evaluation of the mobile health care service in West Kiang district. World Health Statistics Quarterly – Rapport Trimestriel de Statistiques Sanitaires Mondiales.

[B34] Fox-Rushby JA (1995). The Gambia: Cost and effectiveness of a mobile maternal health care service, West Kiang. World Health Statistics Quarterly.

[B35] Fox-Rushby JA, Foord F (1996). Costs, effects and cost-effectiveness analysis of a mobile maternal health care service in West Kiang, The Gambia. Health Policy.

[B36] Xu Z (1995). China: lowering maternal mortality in Miyun County, Beijing. World Health Statistics Quarterly – Rapport Trimestriel de Statistiques Sanitaires Mondiales.

[B37] Zhang T, Wu YQ, Zhang X, Xiong Q, Wang YP, Zhao GL (2004). An evaluation of effects of intervention on maternal and child health in the rural areas of China. Sichuan da Xue Xue Bao Yi Xue Ban/Journal of Sichuan University Medical Science Edition.

