# Supplementary material for: Systematic review of effect of community-level interventions to reduce maternal mortality
Source: BMC Pregnancy Childbirth. 2009 Jan 20;9:2. doi: 10.1186/1471-2393-9-2 (PMC2637835; doi:10.1186/1471-2393-9-2)
Supplement: Additional file 1 — Appendix. Search strategy used in systematic review of community-level interventions to reduce maternal mortality [file 1471-2393-9-2-S1.doc]

Appendix: search strategy used in systematic review of community-level interventions to reduce maternal mortality

MEDLINE

1 randomized controlled trial.pt.

2 controlled clinical trial$.pt.

3 randomized controlled trials.sh.

4 random allocation.sh.

5 double blind method.sh.

6 single blind method.sh.

7 or/1-6

8 (animals not human).sh.

9 7 not 8

10 clinical trial.pt.

11 exp clinical trials/

12 ((singl$ or doubl$ or trebl$ or tripl$) adj25 (blind$ or mask$)).ti,ab.

13 placebo$.ti,ab.

14 random$.ti,ab.

15 placebo$.sh.

16 (clin$ adj25 trial$).ti,ab.

17 research design.sh.

18 or/10-17

19 18 not 8

20 19 not 9

21 comparative study.sh.

22 exp evaluation studies/

23 follow up studies.sh.

24 prospective studies.sh.

25 (control$ or prospective$ or volunteer$).ti,ab.

26 or/21-25

27 26 not 8

28 26 not (9 or 20)

29 9 or 20 or 28

30 cohort study.sh.

31 cohort studies.sh.

32 31 not 8

33 32 not 29

34 33 or 9 or 20 or 29

35 self help group$.mp. or exp self help groups/

36 exp midwifery/

37 exp maternal health services/ or exp postnatal care/ or exp preconception care/ or exp prenatal care/

38 exp nurse midwives/

39 (birth$ adj2 attend$).mp. [mp=title, original title, abstract, name of substance word, subject heading word]

40 (lay midwife$ or lay midwive$).mp. [mp=title, original title, abstract, name of substance word, subject heading word]

41 (traditional midwife$ or traditional midwive$).mp. [mp=title, original title, abstract, name of substance word, subject heading word]

42 (community midwife$ or community midwive$).mp. [mp=title, original title, abstract, name of substance word, subject heading word]

43 (education$ adj4 interven$).mp. [mp=title, original title, abstract, name of substance word, subject heading word]

44 (participat$ adj4 interven$).mp. [mp=title, original title, abstract, name of substance word, subject heading word]

45 (commun$ adj4 interven$).mp.

46 or/35-45

47 (Maternal adj4 mortal$).mp. or exp Maternal Mortality/

48 (maternal adj4 death$).mp.

49 (pregnan$ adj4 mortal$).mp.

50 (pregnan$ adj4 death$).mp.

51 or/47-50

52 51 and 46 and 34

53 51 and 46 and 29

EMBASE

1 randomized controlled trial$.pt.

2 controlled clinical trial$.pt.

3 randomized controlled trial$.sh.

4 random allocation.sh.

5 double blind method.sh.

6 single blind method.sh.

7 or/1-6

8 clinical trial.pt.

9 exp clinical trials/

10 (clin$ adj25 trial$).ti,ab.

11 ((singl$ or doubl$ or trebl$ or tripl$) adj25 (blind$ or mask$)).ti,ab.

12 placebo$.ti,ab,sh.

13 random$.ti,ab,sh.

14 research design.sh.

15 exp clinical trial/

16 exp Randomized controlled trial/

17 exp double blind procedure/

18 exp single blind procedure/

19 exp randomization

20 exp clinical trial/

21 or/9-20

22 (comparative study or comparative studies).sh.

23 exp evaluation studies/

24 follow up studies.sh.

25 prospective studies.sh.

26 (control$ or prospective$ or volunteer$).ti,ab.

27 or/22-26

28 cohort studies.sh.

29 cohort study.sh.

30 exp Cohort Analysis/

31 21 or 27 or 30

32 30 not (21 or 7)

33 limit 31 to human

34 limit 32 to human

35 (education$ adj2 intervention$).mp. [mp=title, abstract, subject headings, heading word, drug trade name, original title, device manufacturer, drug manufacturer name]

36 (commun$ adj2 interven$).mp. [mp=title, abstract, subject headings, heading word, drug trade name, original title, device manufacturer, drug manufacturer name]

37 (participat$ adj2 interven$).mp. [mp=title, abstract, subject headings, heading word, drug trade name, original title, device manufacturer, drug manufacturer name]

38 self help group$.mp.

39 exp self help/

40 (birth$ adj2 attend$).mp. [mp=title, abstract, subject headings, heading word, drug trade name, original title, device manufacturer, drug manufacturer name]

41 (lay midwife$ or lay midwive$).mp.

42 community health worker$.mp.

43 exp pregnancy/ or exp prenatal care/ or antenatal care.mp.

44 or/35-43

45 exp Maternal Mortality/

46 (Maternal adj4 mortality).mp.

47 (maternal adj4 death$).mp.

48 (pregnan$ adj4 death$).mp.

49 (pregnan$ adj4 mortality$).mp.

50 or/45-49

51 50 and 44 and 33

52 50 and 44 and (27 or 21)

53 limit 52 to human

54 or/35-42

55 or/1-30

56 55 and 54 and 50

57 limit 56 to human

CINAHL

1 exp Support Groups/

2 exp maternal health services/ or exp postnatal care/ or exp prenatal care/ or exp prepregnancy care/

3 exp Lay Midwifery/ or exp Lay Midwives/ or birth attendant$.mp.

4 (education$ adj2 intervention$).mp. [mp=title, subject heading word, abstract, instrumentation]

5 (commun$ adj2 interven$).mp. [mp=title, subject heading word, abstract, instrumentation]

6 (participat$ adj2 interven$).mp. [mp=title, subject heading word, abstract, instrumentation]

7 community health worker$.mp. or exp Community Health Workers/

8 self help group$.mp. or exp Support Groups/

9 antenatal care.mp.

10 (birth$ adj2 attend$).mp.

11 or/1-10

12 maternal mortality.mp. or exp Maternal Mortality/

13 (maternal adj4 mortality).mp.

14 (maternal adj4 death$).mp. [mp=title, subject heading word, abstract, instrumentation]

15 (pregnan$ adj4 mortality$).mp.

16 (pregnan$ adj2 death$).mp. [mp=title, subject heading word, abstract, instrumentation]

17 or/12-16

18 11 and 17

19 limit 18 to research

20 randomized controlled trial$.mp. [mp=title, subject heading word, abstract, instrumentation]

21 exp Clinical Trials/

22 exp Prospective Studies/mt, ev [Methods, Evaluation]

23 exp Random Assignment/ or exp Clinical Trials/ or exp Random Sample/ or randomization.mp. or exp Research Methodology/

24 randomized controlled trial$.pt.

25 controlled trial.pt.

26 double blind method.sh.

27 single blind method.sh.

28 clinical trial.pt.

29 exp Clinical Trials/

30 (clin$ adj25 trial$).ti,ab.

31 ((singl$ or doubl$ or trebl$ or tripl$) adj25 (blind$ or mask$)).ti,ab.

32 placebo$.ti,ab,sh.

33 random$.ti,ab,sh.

34 or/20-33

35 (comparative study or comparative studies).sh.

36 exp Evaluation Research/

37 follow up studies.sh.

38 exp Prospective Studies/ec, mt, ev [Economics, Methods, Evaluation]

39 (control$ or prospective$ or volunteer$).ti,ab.

40 or/36-39

41 cohort studies.mp.

42 34 or 40 or 41

43 42 and 11 and 17

44 43 not 19

45 19 not 44

46 19 not 43

47 19 or 43

BNI

1 (education$ adj4 interven$).mp. [mp=heading words, title]

2 (community adj4 intervention$).mp. [mp=heading words, title]

3 (participatory adj4 intervention$).mp. [mp=heading words, title]

4 (self-help groups$ or self help group$).mp. [mp=heading words, title]

5 (midwife$ or midwive$).mp. [mp=heading words, title]

6 prenatal care.mp. [mp=heading words, title]

7 postnatal care.mp. [mp=heading words, title]

8 preconception care.mp. [mp=heading words, title]

9 perinatal care.mp. [mp=heading words, title]

10 (evaluation study or evaluation studies).mp. [mp=heading words, title]

11 follow up.mp. [mp=heading words, title]

12 (follow up study or follow up studies).mp. [mp=heading words, title]

13 intervention$.mp. [mp=heading words, title]

14 (prospective study or prospective studies).mp. [mp=heading words, title]

15 (control$ or prospective$ or volunteer$).mp. [mp=heading words, title]

16 trial$.mp. [mp=heading words, title]

17 randomized controlled trial$.mp. [mp=heading words, title]

18 controlled trial$.mp. [mp=heading words, title]

19 exp randomized controlled trials/

20 (cluster adj4 trial$).mp. [mp=heading words, title]

21 exp clinical trials/

22 or/16-21

23 community health worker$.mp. [mp=heading words, title]

24 (birth$ adj2 attend$).mp. [mp=heading words, title]

25 (cohort studies or cohort study).mp.

26 (intervention study or intervention studies).mp. [mp=heading words, title]

27 (compar$ study or compar$ studies).mp. [mp=heading words, title]

28 (clin$ adj25 trial$).ti,ab.

29 ((singl$ or doubl$ or trebl$ or tripl$) adj25 (blind$ or mask$)).ti,ab.

30 placebo$.ti,ab,sh.

31 random$.ti,ab,sh.

32 research design.sh.

33 or/25-32

34 or/10-21

35 33 or 34

36 or/1-9

37 36 or 23 or 24

38 (Maternal adj4 mortality).mp.

39 (Maternal adj4 death$).mp.

40 (pregnan$ adj4 death$).mp. [mp=heading words, title]

41 (pregnan$ adj4 mortality).mp.

42 or/38-41

43 42 and 37 and 35

44 [from 43 keep 1-4]

45 36 and 42

46 or/10-21

47 or/25-32

48 46 or 47

49 45 and 48

IBSS

- - 1. (education$ adj4 interven$).mp. [mp=abstract, title, subject heading, geographic heading]
    2. (community adj4 intervention$).mp. [mp=abstract, title, subject heading, geographic heading]
    3. (participatory adj4 intervention$).mp. [mp=abstract, title, subject heading, geographic heading]
    4. (self-help groups$ or self help group$).mp. [mp=abstract, title, subject heading, geographic heading]
    5. (evaluation study or evaluation studies).mp. [mp=abstract, title, subject heading, geographic heading]
    6. follow up.mp. [mp=abstract, title, subject heading, geographic heading]
    7. (follow up study or follow up studies).mp. [mp=abstract, title, subject heading, geographic heading]
    8. intervention$.mp. [mp=abstract, title, subject heading, geographic heading]
    9. (prospective study or prospective studies).mp. [mp=abstract, title, subject heading, geographic heading]
    10. (control$ or prospective$ or volunteer$).mp. [mp=abstract, title, subject heading, geographic heading]
    11. trial$.mp. [mp=abstract, title, subject heading, geographic heading]
    12. randomized controlled trial$.mp. [mp=abstract, title, subject heading, geographic heading]
    13. controlled trial$.mp. [mp=abstract, title, subject heading, geographic heading]
    14. [exp randomized controlled trials/]
    15. (cluster adj4 trial$).mp. [mp=abstract, title, subject heading, geographic heading]
    16. [exp clinical trials/]
    17. or/16-21
    18. community health worker$.mp. [mp=abstract, title, subject heading, geographic heading]
    19. (birth$ adj2 attend$).mp. [mp=abstract, title, subject heading, geographic heading]
    20. (cohort studies or cohort study).mp.
    21. (intervention study or intervention studies).mp. [mp=abstract, title, subject heading, geographic heading]
    22. (compar$ study or compar$ studies).mp. [mp=abstract, title, subject heading, geographic heading]
    23. (clin$ adj25 trial$).ti,ab.
    24. ((singl$ or doubl$ or trebl$ or tripl$) adj25 (blind$ or mask$)).ti,ab.
    25. placebo$.ti,ab,sh.
    26. random$.ti,ab,sh.
    27. research design.sh.
    28. or/25-32
    29. or/10-21
    30. 33 or 34
    31. or/1-9
    32. 36 or 23 or 24
    33. (Maternal adj4 mortality).mp.
    34. (Maternal adj4 death$).mp.
    35. (pregnan$ adj4 death$).mp. [mp=abstract, title, subject heading, geographic heading]
    36. (pregnan$ adj4 mortality).mp.
    37. or/38-41
    38. 42 and 37 and 35

CAB abstracts:

1 randomized controlled trial$.sh.

2 randomi$ controlled trial$.mp. [mp=abstract, title, original title, broad terms, heading words]

3 (doubl$ adj blind$).mp. [mp=abstract, title, original title, broad terms, heading words]

4 clinical trial$.mp. [mp=abstract, title, original title, broad terms, heading words]

5 random$.ti,ab,sh.

6 placebo$.ti,ab,sh.

7 ((singl$ or doubl$ or trebl$ or tripl$) adj25 (blind$ or mask$)).ti,ab.

8 (compar$ study or compar$ studies).mp.

9 (cohort studies or cohort study).mp.

10 (control$ or prospective$ or volunteer$).mp.

11 trial$.mp.

12 (cluster adj4 trial$).mp.

13 (evaluat$ study or evaluat$ studies).mp.

14 or/1-13

15 (education$ adj2 intervention$).mp. [mp=abstract, title, original title, broad terms, heading words]

16 (commun$ adj2 interven$).mp. [mp=abstract, title, original title, broad terms, heading words]

17 (participat$ adj2 interven$).mp. [mp=abstract, title, original title, broad terms, heading words]

18 self help group$.mp.

19 (midwife$ or midwive$).mp. [mp=abstract, title, original title, broad terms, heading words]

20 preconception care.mp.

21 prenatal care.mp.

22 antenatal care.mp. [mp=abstract, title, original title, broad terms, heading words]

23 perinatal care.mp.

24 postnatal care.mp.

25 (birth$ adj4 attend$).mp. [mp=abstract, title, original title, broad terms, heading words]

26 community health worker$.mp. [mp=abstract, title, original title, broad terms, heading words]

27 or/15-26

28 (pregnan$ adj4 death$).mp.

29 (pregnan$ adj4 mortal$).mp.

30 maternal mortality.mp. [mp=abstract, title, original title, broad terms, heading words]

31 (maternal adj4 death$).mp. [mp=abstract, title, original title, broad terms, heading words]

32 (maternal adj4 mortal$).mp. [mp=abstract, title, original title, broad terms, heading words]

33 or/28-32

34 33 and 27 and 14

**Cochrane** Central, DARE, NHS EED and Systematic reviews:

#1 (education* near intervention*):ti,ab,kw

#2 (communit* near intervention*):ti,ab,kw

#3 (participat* near intervention*):ti,ab,kw

#4 “self help group”

#5 (birth near attendant*)

#6 MeSH descriptor Maternal Health Services explode all trees

#7 MeSH descriptor Midwifery explode all trees

#8 #1 or #2 or #3 or #4 or #5 or #6 or #7

#9 (pregnan* near (mortalit* or death*))

#10 (maternal near (mortalit* or death*))

#11 MeSH descriptor Maternal Mortality explode all trees

#12 #9 or #10 or #11

#13 #8 and #12

# ISI Web of Science: SCI-EXPANDED, SSCI

1. TS=(maternal mortalit* or maternal death* or pregnan* mortalit* or pregnan* death*)
2. TS=(education* intervention* or communit* intervention* or participat* intervention*)
3. TS=(self help group*or self-help group*)
4. TS=(maternal health service* or prenatal care or perinatal care or postnatal care)
5. TS=(birth attendant*)
6. TS=(midwife* or midwive*)
7. #6 OR #5 OR #3 OR #2
8. TS=(controlled trial* or randomized controlled trial* or random allocation or double blind method or single blind method)
9. TS=(singl*blind* or double blind* or tripl* blind*or trebl* blind*)
10. TS=(clinical trial*)
11. TS=(placebo* or random* or research design)
12. TS=(comparative study or comparative studies or comparison study or comparison studies)
13. TS=(evaluation or follow-up or prospective or volunteers)
14. #13 OR #12 OR #11 OR #10 OR #9 OR #8
15. #14 AND #7 AND #1
16. TS=(birth* attend*)
17. TS=(community health worker)
18. #17 OR #16 OR #4 OR #3 OR #2
19. #18 AND #14 AND #1

**and**

ISI Web of Science

1. TS=(maternal mortalit* or maternal death* or pregnan* mortalit* or pregnan* death*)
2. TS=(randomized controlled trial*)
3. #2 AND #1

**LILACS:**

"MATERNAL MORTALITY" or "MATERNAL MORTALITY/" or "MATERNAL PATTERNS OF CARE" or "MATERNAL PATTERNS OF CARE/" or "MATERNAL WELFARE/" [Palavras]

 and

 "TRIAL" or "TRIALS" or "RANDOM" or "RANDOM ALLOCATION" or "RANDOM ALLOCATION/" or "RANDOM AND SYSTEMATIC SAMPLING" or "RANDOM AND SYSTEMATIC SAMPLING/" or "RANDOMISED" or "RANDOMIZED" or "RANDOMIZED CONTROLLED TRIALS" or "RANDOMIZED CONTROLLED TRIALS/" or "CLUSTER ANALYSIS" or "CLUSTER ANALYSIS/" or "INTERVENTION STUDIES" or "INTERVENTION STUDIES/" or "COHORT" or "COHORT ANALYSIS" or "COHORT ANALYSIS/" or "COHORT STUDIES" or "COHORT STUDIES/" or "PROSPECTIVE" [Palavras]

**Afican Index Medicus:**

"RANDOMISED" or "RANDOMIZED" or "TRIAL" or "TRIALS" or "STUDIES" or "STUDY" or "INTERVENTION" or "INTERVENTIONAL" or "INTERVENTIONS" or "COHORT" [Key Word]

and

 "MIDWIFE" or "MIDWIFERY" or "MIDWIVES" or "BIRTH" or "MATERNITY" or "PREGNANCIES" or "PREGNANCY" or "PREGNANT" or "MATERNITY" or "MATERNAL" or "MATERNALLY" or "MATERNITY" or "ANTENATAL" or "POSTNATAL" or "POSTPARTUM" or "PRENATAL" [Key Word]
